# Surgical options for correction of refractive error following cataract surgery

**DOI:** 10.1186/s40662-014-0002-2

**Published:** 2014-10-14

**Authors:** Ahmed A Abdelghany, Jorge L Alio

**Affiliations:** Clinical research fellow in Vissum Corporación Alicante, Universidad Miguel Hernández, Alicante, Spain; Minia University, Minia, Egypt; Vissum Corporación, Alicante, Spain; Division of Ophthalmology, Universidad Miguel Hernández, Alicante, Spain; Avda de Denia s/n, Edificio Vissum, Alicante, 03016 Spain

**Keywords:** Cataract surgery, Target refraction, Residual refractive error, Refractive surprise, Excimer laser surgery, Photorefractive keratectomy, Intraocular lens exchange, Piggyback lens

## Abstract

Refractive errors are frequently found following cataract surgery and refractive lens exchange. Accurate biometric analysis, selection and calculation of the adequate intraocular lens (IOL) and modern techniques for cataract surgery all contribute to achieving the goal of cataract surgery as a refractive procedure with no refractive error.

However, in spite of all these advances, residual refractive error still occasionally occurs after cataract surgery and laser in situ keratomileusis (LASIK) can be considered the most accurate method for its correction. Lens-based procedures, such as IOL exchange or piggyback lens implantation are also possible alternatives especially in cases with extreme ametropia, corneal abnormalities, or in situations where excimer laser is unavailable. In our review, we have found that piggyback IOL is safer and more accurate than IOL exchange.

Our aim is to provide a review of the recent literature regarding target refraction and residual refractive error in cataract surgery.

**Electronic supplementary material:**

The online version of this article (doi:10.1186/s40662-014-0002-2) contains supplementary material, which is available to authorized users.

## 1Introduction

Cataract removal with Intraocular lens (IOL) implantation is one of the most frequently performed surgical procedures in current clinical practice. Modern microsurgical techniques, new IOL technologies, sophisticated biometry methods, and advanced methods of IOL power calculation allow most cataract patients to regain high-quality vision. The main issue to avoid refractive surprise following cataract surgery is the accuracy of the IOL calculation together with the selection of the appropriate biometric formula for each case.

Indications for cataract surgery have increased as a result of the excellent outcomes and the high predictability of the technique. Modern cataract surgery is a refractive procedure and is performed to correct a refractive error such as myopia, hyperopia and astigmatism especially when associated to a decrease in accommodation [1]-[3].

Advances in small incision surgery have enabled cataract surgery to develop from being concerned primarily with the safe removal of the opaque crystalline lens into a procedure refined to yield the best possible postoperative refractive result [4].

Such success has further promoted the indication of refractive lens exchange (RLE). The outcomes of RLE in myopia and hyperopia and the frequency of secondary refractive procedures needed in different series can be seen in (Tables 1 and 2) [5]-[10].

**Table 1:** **Outcomes of refractive lens exchange (RLE) in myopia and frequency of secondary refractive procedure needed**

	Number of patients	Number of eyes	Mean age (years)	Formula	Before RLE mean SE (D)	After RLE mean SE (D)	Efficacy	Predictability	Follow up	Secondary refractive procedure (after RLE)
Vicary (1999) [5]	42	42	48.9 (range 22–69)	SRK-T	−2.9 ± 7.72 (range −0.25 to −23.75)	−0.23 (±1.08)	91.6% (±1.00 D)	NR	Range 2-26 months	16.67% (7 eyes) - piggyback IOL (3 eyes) - IOL exchange (4 eyes)
Jose Guell (2003) [6]	30	44	42.8 (range 30–49)	SRK-T	−15.77 (range −3.5 to −29)	−1.05 (range +2.75 to −4.75)	52.7% (±1.00 D) 94.1% (±2.00 D)	NR	Range 21–53 months	9.09% (4 eyes) - LASIK (2 eyes) - AK (2 eyes)
Horgan (2005) [7]	37	62	45.3 (±9)	NR	−13.7 (±4.3) (range −7.00 to - 22.75)	−1.09 (±1.34) (range +2.00 to −5.375)	NR	NR	Range 9 months to 10 years	NR
Joao Arraes (2006) [8]	35	60	50.3 (±10.7)	NR	−17	−1.7	NR	NR	20 months	NR
Luis F. Vega (2007) [9]	33	60	Range (45–70)	SRK-T	−5.56 ± 2.82 (range −0.75 to −11)	+0.19 ± 0.37	97% (±1.00 D)	90.9% (±0.50 D)	6 months	no

SE = spherical equivalent, RLE = refractive lens exchange, IOL = intraocular lens, LASIK = laser in situ keratomileusis, AK = arcuate keratotomy, NR = not reported.

**Table 2:** **Outcomes of refractive lens exchange (RLE) in hyperopia and frequency of secondary refractive procedure needed**

	Number of patients	Number of eyes	Mean age (years)	Formula	Before RLE mean SE (D)	After RLE mean SE (D)	Efficacy	Predictability	Follow up	Secondary refractive procedure (after RLE)
Fink (2000) [10] A < +5 D B ≥ +5 D	29	A: 26 B: 24	A: 61.9 B: 54.7	Holladay II	A: +2.26 ± 0.94 B: +6.32 ± 1.32	B: −0.18 ± 0.73 B: +0.19 ± 1.28	A: 97.3% B: 98.1%	NR	10 months	14% (7 eyes) - RK (2 eyes) - LTK (2 eyes) - PRK (1 eye) - AK (1 eye) -Sutures (1 eye)
Luis F. Vega (2007) [9]	79	158	Range (45–70)	Holladay II	+3.54 ± 2.49 (range +0.75 to +8.5)	+0.23 ± 0.32	NR	96% (±1 D)	6 months	no

SE = spherical equivalent, RLE = refractive lens exchange, RK = radial keratotomy, LTK = laser thermal keratoplasty, PRK = photorefractive keratectomy, AK = arcuate keratotomy, LASEK = laser-assisted subepithelial keratectomy, NR = not reported.

Some patients also seek spectacle independence for near vision after cataract surgery. In these cases, the use of multifocal lenses provides an alternative for the correction of presbyopia. However, for a multifocal IOL to be efficient, astigmatism must be completely eliminated. A laser touch-up is required if there is residual astigmatism over 1.00 D after the multifocal IOL implantation. Viser et al. reported about 4% of patients having over 3.00 D of corneal astigmatism while 70% of patients having over 1.00 D of corneal astigmatism. The availability to use toric multifocal IOLs is therefore of great importance [11].

Emmetropia is the goal in most cataract cases. However this was achieved in only 55% of eyes in some national series [12].

Despite new advances in cataract surgery, unsatisfactory visual outcome as a result of a residual refractive error occasionally occurs. Refractive surprise after cataract surgery is an unpleasant and frustrating situation for both the patient and the physician.

Different surgical techniques are proposed for the correction of the residual refractive error, these being corneal-based surgery (laser refractive surgery) and lens-based procedures (IOL exchange or piggyback IOLs) [13].

## 2Review

### 2.1 Target refraction (Is emmetropia the only target refraction in all cataract cases?)

Emmetropia (spherical equivalent −0.5 to +0.5 D and <1.0 D astigmatism) is the target refraction in most cataract cases. The ability to achieve target refraction may be a good objective and a quantitative indicator of surgical outcomes. However, it must be taken into account that technically good surgical results can also be associated with poor postoperative visual acuity in patients whose vision is limited by retinal or optic nerve disease [14].

The most important factors to achieve the target refraction are the accuracy of the IOL calculation along with an appropriate choice of the surgical procedure. Over the past decades the main focus has been on improving IOL power calculation formulas. Despite significant improvements, IOL power calculation formulas still generally work best in eyes with a normal axial length (AL) while biometry prediction errors tend to increase in long eyes [15] and in short eyes [16] with the commonly used formulas.

Cataract surgery does not necessarily aim to achieve a postoperative refractive error with no astigmatism. A “physiological” astigmatism of up to 1.0 D either with or against the rule may be useful to increase the depth of focus under routine conditions, and may increase the quality of vision in daily life. Astigmatism of up to 1.0 D may also be considered as a physiological measure to reduce uncorrected presbyopia for eyes with intact retina and optic nerve [17].

If the aim of the cataract surgery is to achieve best uncorrected distance and near vision, the target refraction should be in the range of −1.0 D to −1.5 D [17].

However, even with this refraction, a satisfactory uncorrected visual acuity for distance and near vision cannot be achieved on a regular basis with unilateral cataract surgery combined with the implantation of monofocal IOLs. This raises the question of whether satisfactory uncorrected distance and near vision can be achieved by bilateral cataract surgery with induced monovision, in which one eye has a postoperative refraction of about −1.75 D and the contralateral eye close to emmetropia. An alternative would be the implantation of multifocal IOLs or other techniques of corneal refractive surgery such as the use of corneal inlays in presbyopic patients after laser-assisted in situ keratectomy [18]-[20].

### 2.2 Causes of refractive surprise following cataract surgery

The most frequent complication following cataract surgery is residual refractive error resulting in suboptimal visual outcomes, and this may be due to preoperative, operative or postoperative causes.

Preoperative causes include misestimation of postoperative IOL position and preoperative AL measurement [21], inadequate selection of the IOL power, limitations of the calculation formulas (especially in extreme ametropia) and the lack of precision in the manufacturing of IOLs. In addition, there is a potential error in the prediction of the postoperative anterior chamber depth by the current IOL power formulas [21].

Another limiting factor for optimum uncorrected postoperative visual acuity is preexisting corneal astigmatism, which is reported to exceed 1.00 D in approximately a third of cataract patients with some variations between populations [22],[23].

There are some risk groups with difficult IOL calculation and refractive surprise after cataract surgery is possible in these cases, such as in patients with previous laser in situ keratomileusis (LASIK), photorefractive keratectomy (PRK), radial keratotomy (RK) and patients with keratoconus due to corneal surface irregularity.

Patients who have previously undergone myopic correction either by LASIK or PRK may have an undesirable hyperopic refractive result following cataract surgery due to errors in estimating corneal power and effective lens position, which are two important variables in IOL calculation. The change in the optical profile of the cornea and the existence of variable degrees of regular and/or irregular astigmatism are common in all corneal refractive procedures [24].

Operative causes include surgical variations in the size and central position of the capsulorhexis, which may influence the final position of the IOL inside the bag and are surgeon-dependent. Surgically induced astigmatism may also be a cause of refractive error after cataract surgery [25].

Residual astigmatism following cataract surgery with toric IOL implantation has been reported and may be due to the effect of the spherical power and anterior chamber depth in toric IOL calculations, the effect of posterior corneal astigmatism, and the effect of a large pupil size. The first two issues may be compensated for by improving toric IOL calculations. The latter indicates that pupillometry is required in relatively young patients who undergo toric IOL implantation [26].

Postoperative causes may occur during the healing process such as anterior movement of the IOL resulting from postoperative capsular bag fibrosis and contraction. Studies have shown mean myopic shifts in spherical equivalent refraction of 0.70 D from 1 day postoperatively up to 2 months [27].

### 2.3 How we can avoid refractive surprise following cataract surgery?

Modern microsurgical techniques, new IOL technologies, sophisticated biometry methods, and advanced methods of IOL power calculation allow most cataract patients to regain high-quality vision.

Accurate IOL calculation is important to avoid refractive surprise especially in certain cases, such as cases with previous refractive corneal surgery in which previous refractive surgery data is often unavailable and even when available, may be based on measurements taken with the technology used many years ago.

Yang et al. have compared eight methods for IOL power calculation for post-myopic excimer laser surgery patients without previous refractive surgery data, and concluded that the Holladay 2 Flat K method provided the most accurate IOL power in these patients. If the Holladay IOL Consultant Program is unavailable, the ASCRS methods can be used, the ASCRS-Min which is the most accurate method [28].

In order to minimize surgically induced astigmatism (SIA), micro incision cataract surgery (MICS) using corneal topography data and standard formulas for the calculation of the IOL power is a safe and effective procedure in terms of keratometric stability, visual and refractive results [29]. A secure, watertight sub 2.2 mm clear corneal micro incision is recommended, which is located optimally less than 1 mm from the limbus and situated on the steepest corneal meridian in order to minimize surgically induced astigmatism or to intentionally reduce pre-existing corneal astigmatism.

In addition to the well-known influence of incision size on SIA, corneal hysteresis also modulates optical changes. The biomechanical features of the cornea should be taken into account preoperatively to better predict the refractive outcomes of cataract surgery [30].

Cataract surgeons should consider ways of dealing with preexisting corneal astigmatism when aiming for emmetropia. Approaches to reduce corneal astigmatism include incision placement on the steep axis, peripheral corneal relaxing incisions, and implantation of a toric IOL [12].

We can benefit from residual refractive error in patients undergoing bilateral sequential cataract surgery in which the refractive error in the first eye exceeds 0.50 D; in these cases the refractive error in the second eye can be improved by modifying the IOL power [31].

### 2.4 Surgical options for correction of refractive error following cataract surgery

It is important to know the different methods that can be used to resolve refractive surprise after cataract surgery, such as corneal-based surgery (laser refractive surgery) and lens-based procedures (IOL exchange or piggyback IOLs) and the expected outcomes and possible complications of the different procedures.

#### 2.4.1 Lens-based procedures (IOL exchange or piggyback IOLs)

Lens based procedures are preferable in some situations and have certain advantages [32].

1. If correction of the residual refractive error is desired soon after cataract surgery, the original cataract wound can be reopened and the IOL implanted soon after the initial surgery (IOL exchange).
2. If there is a large postoperative refractive surprise, lens based procedures are effective in reducing high degrees of spherical error.
3. Lens based procedures do not alter the anterior corneal surface and do not significantly change the corneal refractive power.
4. There is no need for special settings such as those required for laser refractive surgery.

**Figure 1:**
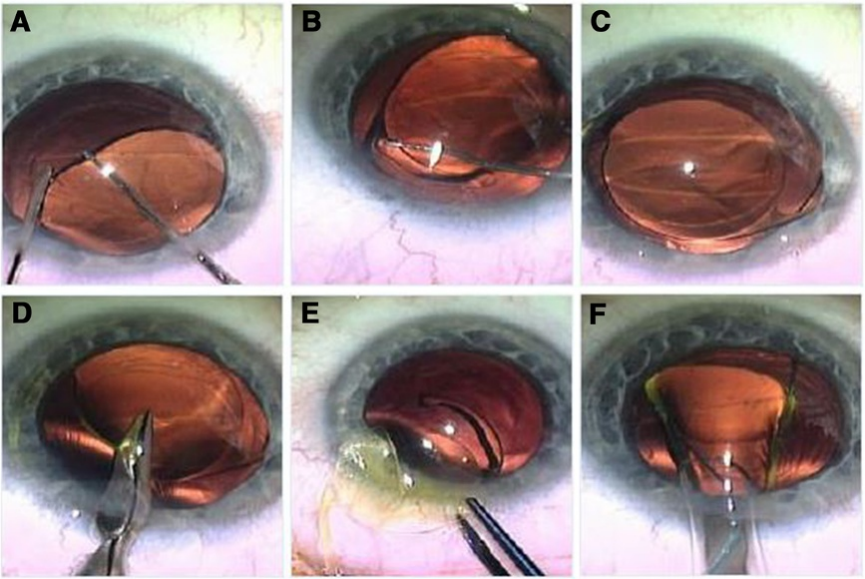
**IOL explantation.** Opening of the same corneal incision of the previous cataract surgery and paracentesis, injection of viscoelastic and freeing of the optic of IOL from the capsular bag **(A)**. Dialing of the first haptic to explant it outside the capsular bag **(B)**, and then dialing of the IOL to explant the whole IOL outside the capsular bag **(C)**. Cutting part of the IOL optic with scissors for easy explantation through the small wound **(D)**. Explantation of IOL haptic, then the optic, then the other haptic through the same small wound without widening it **(E)**. Implantation of the 2^nd^ IOL by injector through the main wound **(F)**.

It has been reported that incorrect power is the second most frequent indication for IOL exchange. If the lens to be removed is foldable it can be cut and removed through a small incision (Figure 1) [33].

The piggyback technique involves the implantation of two IOLs in the posterior chamber of the same eye. It is easier than exchanging the original IOL as sometimes the original IOL is strongly adherent to the capsular bag and its removal may cause rupture of the capsular bag and zonular damage, which may lead to cyclodialysis, retinal tears and macular edema [32].

Piggyback IOLs have been reported to be more accurate than IOL exchange [31]. With piggyback IOL implantation it is not necessary to know exactly why the residual refractive error occurred. Moreover, the exchanged IOL can be placed in a different plane to the original IOL, which further alters the final refraction, so implantation of a secondary piggyback IOL in the ciliary sulcus that leaves the original IOL in place is an effective, safe and easy treatment for a pseudophakic refractive surprise [32]. Another advantage of a piggyback IOL is its reversibility.

Many types of piggyback IOLs with different designs have been recently used [34],[35].

Basarir et al. implanted the Add-On IOL to correct pseudophakic refractive errors and found that it reduced iris capture, iris trauma and glare effects due to its large optic size and rounded anterior optic edge and also concluded that piggybacking with the Add-On IOL is a safe, efficient and reliable technique to correct residual refractive errors after cataract surgery [34].

Falzon and Stewart implanted the sulcoflex IOL in the ciliary sulcus and also found that it is a predictable option for enhancing postoperative refractive results and reducing spectacle dependence for distance after surgery [35].

Sulcoflex piggyback multifocal IOLs can be used for a hyperopic-presbyopic surprise after cataract surgery in highly myopic patients with the possibility of achieving good near-intermediate visual acuity and spectacle independence, especially in high myopic eyes with good near visual acuity. In addition, the implantation of these IOLs might correct residual refractive errors following previous implantation of a monofocal IOL [36].

Another lens based procedure is the light-adjustable intraocular lens which allows the possibility of correcting postoperative residual refractive errors in a non-invasive way. After implantation and healing, fine-tuning of the refractive power can be performed using ultraviolet light based on the individual requirements of each patient. Up to 2 diopters of sphere, as well as cylinder, can be adjusted in one step [37].

The implantation of a toric IOL could be an interesting option to improve the astigmatic outcome when IOL exchange is planned. The only limitation of this strategy in our view is to accurately estimate the amount of induced astigmatism with the wound enlargement [38].

Considering the above mentioned points, a piggyback IOL is preferable to IOL exchange for the treatment of residual refractive errors after cataract surgery because it is a safe, reversible and more accurate technique [32].

#### 2.4.2 Corneal-based surgery (laser refractive surgery or astigmatic keratotomy)

Laser refractive surgery avoids additional intraocular surgical procedures, provides better accuracy than IOL exchange or piggyback lens techniques especially for cylinder outcomes and gives higher predictability of results [38].

**Figure 2:**
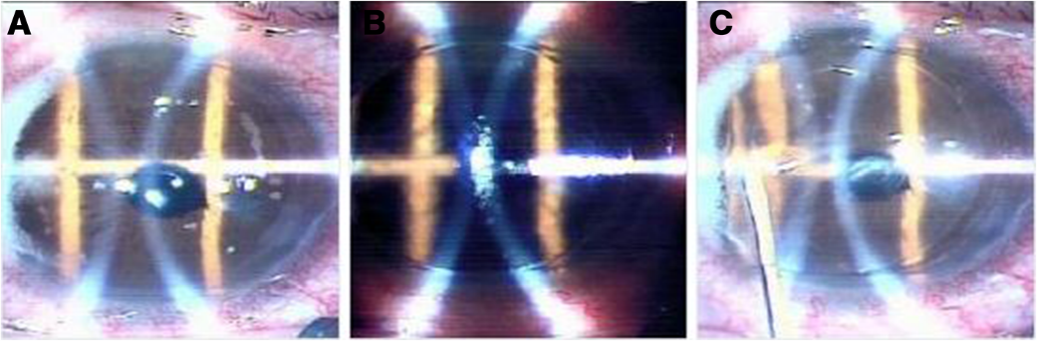
**Lasik on pseudophakic patient (IntraLASIK).** Creation of the flap by femtosecond laser **(A)**, then elevation of the flap and application of excimer laser on the stromal bed **(B)** and finally reposition of the flap and irrigation of the interface **(C)**.

Laser refractive surgery (Figure 2) provides greater flexibility and achieves better final refraction than lens based procedures. In a recent series, LASIK for the correction of residual error after cataract surgery showed that 92.85% of eyes achieved a final spherical equivalent (SE) within ± 0.50 D and 100% of eyes within ± 1.00 D [38].

LASIK seems to be safe in eyes with previous Yttrium aluminium garnet (YAG) capsulotomy. Once YAG capsulotomy has been performed, IOL exchange becomes more difficult and has greater risks.

Once the LASIK flap has been established, additional optical adjustments can be performed successfully whenever necessary [39].

LASIK refinement for the correction of residual refractive error after cataract surgery with monofocal or multifocal IOL implantation is safe and effective, and provides more accurate refractive outcomes in eyes previously implanted with monofocal IOLs [40]. There is a limitation to the predictability of hyperopic LASIK refinement in eyes previously implanted with multifocal IOLs, and this may be because of the existence of errors in the estimation of residual refraction in eyes with multifocal IOLs due to the presence of several foci as well as the estimation of refraction after LASIK. This could be responsible for artifacts in the subjective refraction due to several refractive options providing a similar visual acuity. A reference point for spherical subjective refraction should be established when refracting patients with multifocal IOLs, such as the midpoint of the clear vision interval provided by the depth of field of the IOL in order to avoid postoperative problems of predictability. This midpoint can be easily determined by first defining the range of spherical lenses subjectively providing the same visual quality and the maximum visual acuity (adding and subtracting positives to the first point providing the best visual acuity) and then, considering the lens corresponding to half of this range as the magnitude of the spherical correction. If the same reference point for refraction is always used, the clinician can avoid variability in the achieved spherical correction [40].

It has been reported that wavefront-guided treatments with iris registration may provide better outcomes than conventional LASIK [41],[42]. However, some authors have expressed concern about the accuracy of Shack-Hartmann aberrometers in eyes with multifocal IOLs [43],[44].

Jendritza et al. [45] evaluated the outcomes of wavefront-guided treatment with iris registration after implantation of different multifocal IOLs in 27 eyes of 19 patients and they found good results with diffractive multifocal IOLs but not with refractive multifocal IOLs.

Laser procedures have been reported to be effective and predictable for those desiring monovision after cataract surgery [39].

However, in spite of all these advantages, LASIK has some limitations, such as high refractive error, small corneal stromal thickness and limited availability of excimer laser for cataract surgeons.

Patients with low mixed astigmatism often have reasonable unaided visual acuity and spherical equivalent refraction close to zero. Femtosecond laser intrastromal astigmatic keratotomy has been used to create two arcuate non penetrating intrastromal incisions to correct a low amount of mixed astigmatism and achieve spectacle independence and could be considered a safe and minimally invasive alternative for treating such refractions in cases where other surgical procedures are not possible [46].

## 3Conclusion

LASIK has been shown to be a viable, noninvasive and accurate procedure to correct ametropia after cataract extraction with IOL implantation. Lens-based procedures (IOL exchange or piggyback lens implantation) are also possible alternatives. Piggyback IOLs have proved to be technically easier and more accurate than IOL exchange, and are better indicated in those cases with extreme ametropia, corneal abnormalities, or when there is no available excimer laser platform.

## Authors’ original submitted files for images

Below are the links to the authors’ original submitted files for images. Authors’ original file for figure 1 Authors’ original file for figure 2
