# Supplementary figures and images for: Surgical options for correction of refractive error following cataract surgery
Source: Eye Vis (Lond). 2014 Oct 14;1:2. doi: 10.1186/s40662-014-0002-2 (PMC4604120; doi:10.1186/s40662-014-0002-2)

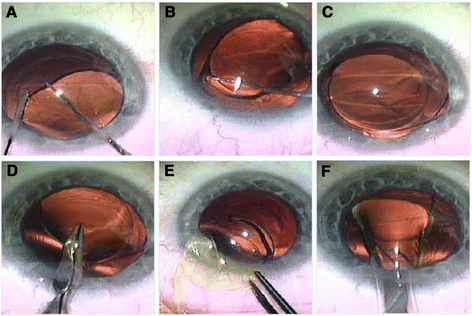

Supplement: Supplementary file 1 — Authors’ original file for figure 1 [file 40662_2014_2_MOESM1_ESM.gif]

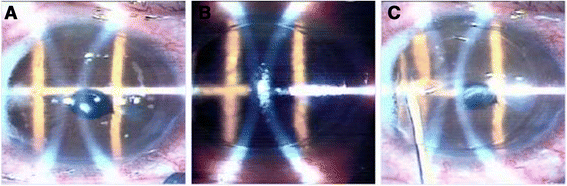

Supplement: Supplementary file 2 — Authors’ original file for figure 2 [file 40662_2014_2_MOESM2_ESM.gif]
